# Fellow-Workers and the Roll of Honour

**Published:** 1914-09-19

**Authors:** 


### Fellow-Workers and the Roll of Honour.

Surgeon-General Arthur Thomas Sloggett, upon whose shoulders falls the responsibility for directing the work of the Army Medical Service during the present war, has had a distinguished military career, in the course of which he has seen much active service. Qualifying L.R.C.P. Ed., M.R.C.S. Eng., in 1880, the Director-General entered the Army Medical Department in the following year, and attained general's rank in 1908. Three years later he became director of the Medical Services in India, and honorary surgeon to His Majesty. This responsible officer's career includes service as senior medical officer to the British troops in the Dongaland expeditionary force in 1896, as a result of which he was mentioned in despatches—receiving promotion and an Egyptian decoration. Subsequently, in the famous Khartoum expedition his horse was on one occasion shot beneath him, whilst he himself was dangerously wounded. South Africa soon followed, with additional distinctions, including the C.M.G.; and after his return home Colonel Sloggett, as he then was, became chief medical officer of the Home and London districts.

Numbers of well-known North Country consultants and practitioners have mobilised with the Northern Military General Hospitals of the Territorial Force. Thus Sir Berkeley Moynihan, a surgeon whose reputation is nowadays world-wide, holds a commission as major in the 2nd Northern unit which has been organised at the Leeds Training College. This eminent surgeon's work in connection with diseases of the stomach and duodenum, and his successful technique for remedying some of the more serious conditions which affect those organs, are well known to every medical man who keeps abreast of current medical literature. He has served the Leeds General Infirmary for many years, and remains a prominent member of the senior surgical staff there. He received the honour of knighthood in 1912.

Dr. A. S. Grünbaum, one of our leading pathologists, is also a major in the 2nd Northern General Hospital. Qualifying from Cambridge and St. Thomas's in 1896, Dr. Grünbaum speedily distinguished himself in pathological work, and has been for some time professor of pathology in the University of Leeds, where he is also Dean of the Medical Faculty. An M.D. of Cambridge, he was made a F.R.C.P. Lond. in 1902.

At Newcastle-on-Tyne many members of the Royal Infirmary's staff have mobilised with the 1st Northern General Hospital, which has been based at the Armstrong College, the laboratories and theatres of which have been fitted up as wards, Dr. T. Beattie, Mr. A. M. Martin, Mr. H. B. Angus, and Dr. W. E. Hume holding the rank of lieutenant-colonel therein.

Dr. Beattie is well known to Newcastle students not only as senior physician to the infirmary, but also as Professor of Therapeutics in Durham University. He is himself a Durham man, whose graduate distinctions included first-class honours and a gold medal.

Mr. A. M. Martin is surgeon and joint lecturer in clinical surgery at the Royal Infirmary, and was at one time assistant surgeon to the Children's Hospital at Newcastle.

Mr. H. B. Angus is also lecturer in surgery at Durham and surgeon to the Royal Infirmary at Newcastle. Qualifying M.R.C.S., L.R.C.P. Lond. in 1881, he subsequently became an M.B. Durh. and F.R.C.S. Eng.

